# Risk of subarachnoid haemorrhage in people admitted to hospital with selected immune-mediated diseases: record-linkage studies

**DOI:** 10.1186/1471-2377-13-176

**Published:** 2013-11-14

**Authors:** Sreeram V Ramagopalan, Julia Pakpoor, Olena Seminog, Raph Goldacre, Lee Graham, Michael J Goldacre

**Affiliations:** 1 Department of Physiology, Anatomy and Genetics and Medical Research Council Functional Genomics Unit, University of Oxford, Oxford, UK; 2 Unit of Health-Care Epidemiology, Department of Public Health, University of Oxford, Oxford, UK

## Abstract

**Background:**

Subarachnoid hemorrhage (SAH) is a devastating cause of stroke, occurring in relatively young people. It has been suggested that some immune-mediated diseases may be associated with an increased risk of SAH.

**Methods:**

We analysed a database of linked statistical records of hospital admissions and death certificates for the whole of England (1999–2011). Rate ratios for SAH were determined, comparing immune-mediated disease cohorts with comparison cohorts.

**Results:**

There were significantly elevated risks of SAH after hospital admission for the following individual immune-mediated diseases: Addison’s disease, ankylosing spondylitis, autoimmune haemolytic anaemia, Crohn’s disease, diabetes mellitus, idiopathic thrombocytopenia purpura, myxoedema, pernicious anaemia, primary biliary cirrhosis, psoriasis, rheumatoid arthritis, scleroderma, Sjogren’s syndrome, SLE and thyrotoxicosis. Elevated risks that were greater than 2-fold were found for Addison’s disease (rate ratio (RR) = 2.01, 95% confidence interval 1.3-2.97), idiopathic thrombocytopenia purpura (RR = 2.42, 1.86-3.11), primary biliary cirrhosis (RR = 2.21, 1.43-3.16) and SLE (RR = 3.76, 3.08-4.55).

**Conclusions:**

Our findings strongly support the suggestion that patients with some immune-mediated diseases have an increased risk of SAH. Further studies of the mechanisms behind this association are warranted.

## Background

Subarachnoid haemorrhage (SAH) accounts for approximately 5% of all strokes [1]. Because it occurs at a relatively young age and has a high case fatality, the loss of productive life years in the general population from SAH is as large as that from other stroke types [1]. The mean age at time of SAH is around 50 years, and the incidence is around 1 per 10,000 people per year, with particularly high rates in Japan and Finland, and higher rates in women than in men [2]. Ruptured aneurysms are the pathological cause in 85% of patients, whereas 10% fit into the pattern of non-aneurysmal perimesencephalic haemorrhage [1]. The remaining 5% have various rare causes [1].

Established personal risk factors for SAH include smoking, hypertension and excessive alcohol intake [2]. Non-modifiable risk factors include a familial preponderance of SAH, female gender and certain heritable connective tissue disorders, such as polycystic kidney disease and Ehlers-Danlos syndrome [2]. In the literature, there have been case reports and case series of patients with immune mediated diseases such as systemic lupus erythematosus (SLE) [3] and Sjogren’s syndrome [4] having SAH, suggesting that there may be a general association between immune-mediated diseases and SAH. In support of this, a recent Swedish study of ischemic and haemorrhagic strokes (but not specifically SAH) in patients hospitalised for a variety of immune-mediated diseases found elevated risks for haemorrhagic stroke in 15 immune-mediated diseases [5].

To investigate this further for SAH, we undertook a record linkage study to determine the risk of SAH in individuals with selected immune-mediated diseases using an English national linked Hospital Episode Statistics (HES) dataset.

## Methods

### Population and data

We used the complete dataset of English national Hospital Episode Statistics (HES) from January 1999 to December 2011, inclusive, covering the population of England of about 50 million people. The HES data were provided by the English national Health and Social Care Information Centre (IC). Data from death registrations, covering the same population and time period, were provided by the Office for National Statistics (ONS). The dataset used in this study, in which successive records for each individual were linked together using personal identifiers irreversibly encrypted by the IC and ONS, was constructed by the Oxford record linkage group (file version fce_14yr_v01_admiyear). Approval for the construction and analysis of the linked dataset was given by the Central and South Bristol Research Ethics Committee (ref 04/Q2006/176).

The basic methods were the same for the analysis of each disease and are described for SLE and SAH. A cohort of people with a record of hospital day case care or inpatient admission for SLE was constructed for those with a principal diagnosis of SLE, as a reason for hospital care, by identifying the first episode of day case care, or inpatient admission, for the condition in an NHS hospital during the study period. The International Classification of Disease (ICD) codes used for each immune-mediated disease are given in Table 1. The ICD codes used for SAH were 430 in the ninth revision of the ICD (the ninth revision was used for mortality data in 1999 and 2000) and I60 in the tenth revision (used for all the hospital data from 1999 and for mortality data from 2001). A reference cohort was constructed by identifying the first admission for each individual with various other, mainly minor medical and surgical conditions and injuries (listed in Table footnotes). This is based on a ‘control’ group of conditions that has been used in other similar studies of associations between diseases [6,7]. In the design of the reference cohort using hospital controls, standard epidemiological practice was followed in selecting a diverse range of conditions, rather than relying on a narrow range (in case the latter are themselves atypical in their risk of subsequent disease). As a check, we have studied the risk of SAH in the control conditions within the reference cohort to ensure that the reference cohort does not include control conditions that have atypically high or low SAH rates.

**Table 1:** Number of people in the study with each immune-mediated disease, and percentage who were female

**Immune-mediated diseases (ICD-10 codes)**	**England, 1999 – 2011**	
	**N**	**% of females**
Addison’s disease (E27)	11 839	59
Ankylosing spondylitis (M45)	29 136	30
Autoimmune haemolytic anaemia (D59.1)	9 241	54
Chronic active hepatitis (K73.2)	4 439	70
Coeliac disease (K90)	70 715	67
Crohn’s disease (K50)	125 240	56
Dermatomyositis (M33.0-M33.1)	2 768	67
Diabetes mellitus (E10)	426 471	46
Goodpasture’s syndrome (M31)	1 058	49
Hashimoto’s thyroiditis (E06.3)	8 630	90
Idiopathic thrombocytopenia purpura (D69)	31 043	55
Multiple sclerosis (G35)	82 256	69
Myasthenia gravis (G70)	11 279	49
Myxoedema (E03.8–E03.9)	938 696	81
Pemphigoid (L12)	13 642	56
Pemphigus (L10)	2 348	56
Pernicious anaemia (D51.0)	67 890	68
Polyarteritis nodosa (M30.0)	2 249	44
Polymyositis (M33.2)	3 860	62
Primary biliary cirrhosis (K74)	11 146	84
Psoriasis (L40)	119 454	48
Rheumatoid arthritis (M05–M06)	331 393	71
Scleroderma (M34)	11 494	82
Sjogren’s syndrome (M35.0)	17 741	89
Systemic lupus erythematosus (M32)	25 576	86
Thyrotoxicosis (E05)	132 826	79
Ulcerative colitis (K51)	180 397	49

People were included in the SLE or reference cohort if they did not have an admission for SAH either before or at the same time as the admission for SLE or the reference condition. We then searched the database for any subsequent NHS hospital care for, or death from, SAH in these cohorts. We considered that rates of SAH in the reference cohort would approximate those in the general population of England while allowing for migration in and out of it (data on migration of individuals were not available).

### Statistical methods

We calculated rates of SAH based on person-days. We took “date of entry” into each cohort as the date of first admission for SLE, or reference condition, and “date of exit” as the date of first record of SAH, death, or the end of data collection (31st December 2011), whichever was the earliest. We first calculated rates for SAH, stratified and then standardised by age (in five-year age groups), sex, calendar year of first recorded admission, region of residence, and quintile of patients’ Index of Deprivation score (as a measure of socio-economic status) [8]. We used the indirect method of standardisation, and the combined SLE and reference cohorts as the standard population. The stratum-specific rates in the combined SLE and reference cohorts were then applied to the number of people in each corresponding stratum in the SLE cohort, and then, separately, to those in the reference cohort, to obtain an ‘expected’ number of people with SAH in each stratum within each of the two cohorts. The stratum-specific observed (O) and expected (E) numbers were then summed. The rate ratio was then calculated, taking the observed and expected numbers of SAH in the SLE cohort relative to those in the reference cohort, using the formula (O^SLE^/E^SLE^) / (O^REF^/E^REF^). The rate ratio, its confidence interval, and χ^2^ statistics for its significance were calculated using standard statistical methods [9]. We have given exact p values so that readers can judge what level of statistical significance each finding has. A full Bonferroni correction for testing multiple comparisons across twenty seven diseases would need an exact p value of 0.00185 to be classed as significant (i.e. p = 0.05/27).

## Results

Table 1 shows the number of people in the study who were admitted to hospital with each of the selected immune-mediated diseases; it also shows the percentage of these who were female. The number of people in the reference cohort was over 7.6 million (48% female).

There were significantly elevated risks of SAH after hospital admission for the following individual immune-mediated diseases: Addison’s disease, ankylosing spondylitis, autoimmune haemolytic anaemia, Crohn’s disease, diabetes mellitus, idiopathic thrombocytopenia purpura, myxoedema, pernicious anaemia, primary biliary cirrhosis, psoriasis, rheumatoid arthritis, scleroderma, Sjogren’s syndrome, SLE and thyrotoxicosis (Table 2).

**Table 2:** **Rate ratios**^**1 **^**and 95% confidence intervals (CIs) for subarachnoid haemorrhage in people with selected immune-mediated diseases, compared with the control cohort**^**2**^

**Immune mediated disease**	**Observed**	**Rate ratio (95% CI)**	**P value**
*Addison’s disease*	*25*	*2.01 (1.3-2.97)*	0.001*
*Ankylosing spondylitis*	*46*	*1.64 (1.2-2.19)*	0.001*
*Autoimmune haemolytic anaemia*	*16*	*1.99 (1.14-3.23)*	*0.009*
Chronic active hepatitis	1	0.18 (0-0.99)	0.08
Coeliac disease	72	1.04 (0.82-1.32)	0.76
*Crohn’s disease*	*159*	*1.29 (1.1-1.51)*	0.001*
Dermatomyositis	3	1.27 (0.26-3.7)	0.93
*Diabetes mellitus*	*534*	*1.21 (1.11-1.33)*	<0.001*
Goodpasture’s syndrome	2	2.31 (0.28-8.34)	0.50
Hashimoto’s thyroiditis	10	0.97 (0.46-1.78)	0.96
*Idiopathic thrombocytopenia purpura*	*62*	*2.42 (1.86-3.11)*	<0.001*
Multiple sclerosis	115	1.09 (0.9-1.31)	0.41
Myasthenia gravis	12	0.93 (0.48-1.62)	0.91
*Myxoedema*	*1283*	*1.15 (1.09-1.22)*	<0.001*
Pemphigoid	16	1.29 (0.74-2.1)	0.38
Pemphigus	3	1.35 (0.28-3.94)	0.85
*Pernicious anaemia*	*96*	*1.3 (1.05-1.59)*	*0.013*
Polyarteritis nodosa	4	1.79 (0.49-4.6)	0.40
Polymyositis	8	1.81 (0.78-3.56)	0.15
*Primary biliary cirrhosis*	*30*	*2.21 (1.49-3.16)*	<0.001*
*Psoriasis*	*156*	*1.34 (1.14-1.57)*	<0.001*
*Rheumatoid arthritis*	*640*	*1.48 (1.36-1.6)*	<0.001*
*Scleroderma*	*25*	*1.84 (1.19-2.72)*	*0.003*
*Sjogren’s syndrome*	*39*	*1.69 (1.2-2.31)*	0.001*
*Systematic lupus erythematosus*	*108*	*3.76 (3.08-4.55)*	<0.001*
*Thyrotoxicosis*	*202*	*1.38 (1.19-1.58)*	<0.001*
Ulcerative colitis	198	1.00 (0.87-1.16)	0.99

^1^Adjusted for sex, age in 5-year bands, time-period in single calendar years, region of residence, and deprivation score associated with patients’ local authority area of residence in quintiles. The rate ratios are calculated as the ratio of the observed/expected number in the cohort for each immune-mediated disease to the observed/expected numbers in the reference cohort as calculated in the analysis undertaken for each of the individual disease (data not shown).

^2^Conditions used in reference cohort: appendicectomy, squint, otitis externa, otitis media, haemorrhoids, deflected nasal septum, nasal polyp, impacted tooth and other disorders of teeth, inguinal hernia, in-growing toenail and other diseases of nail, sebaceous cyst, internal derangement of knee, bunion, dislocations, sprains and strains, superficial injury and contusion.

*Significant P value after full Bonferroni correction = <0.00185.

Levels of risk that were two-fold or higher were found for Addison’s disease (rate ratio (RR) = 2.01, 95% confidence interval 1.3-2.97), idiopathic thrombocytopenia purpura (RR = 2.42, 1.86-3.11), primary biliary cirrhosis (RR = 2.21, 1.43-3.16) and SLE (RR = 3.76, 3.08-4.55).

We studied the occurrence of a first admission for SAH within a year of the first admission for each immune-related disease to help establish whether any elevated risk of SAH was confined to the short-term (and therefore possibly related to the event of admission with the immune-mediated disease) or was more prolonged, although this analysis reduced statistical power. Considering SAH that occurred within a year following the first recorded admission for immune-mediated disease, there were significantly elevated risks for Addison’s disease, ankylosing spondylitis, coeliac disease, diabetes mellitus, idiopathic thrombocytopenia purpura, myxoedema, pernicious anaemia, primary biliary cirrhosis, rheumatoid arthritis, systemic lupus erythematosus and thyrotoxicosis (Table 3).

**Table 3:** **Rate ratios and 95**% **confidence intervals** (**CIs**) **for subarachnoid haemorrhage occurring within a year after admission for immune**-**mediated disease**

**Immune mediated disease**	**Observed**	**Rate ratio (95% CI)**	**P value**
*Addison’s disease*	*7*	*2.77 (1.11-5.73)*	*0.01*
*Ankylosing spondylitis*	*12*	*1.99 (1.02-3.49)*	*0.03*
Autoimmune haemolytic anaemia	5	2.33 (0.75-5.44)	0.11
Chronic active hepatitis	0	0 (0-3.75)	0.63
*Coeliac disease*	*23*	*1.62 (1.03-2.45)*	*0.03*
Crohn’s disease	26	1.16 (0.75-1.71)	0.52
Dermatomyositis	1	1.75 (0.04-9.77)	0.93
*Diabetes mellitus*	*144*	*1.68 (1.40-2.00)*	<0.001*
Goodpasture’s syndrome	0	0 (0-16.34)	0.56
Hashimoto’s thyroiditis	2	0.95 (0.11-3.42)	0.79
*Idiopathic thrombocytopenia purpura*	*22*	*3.72 (2.32-5.66)*	<0.001*
Multiple sclerosis	28	1.47 (0.97-2.14)	0.06
Myasthenia gravis	4	1.44 (0.39-3.71)	0.66
*Myxoedema*	*460*	*1.54 (1.38-1.72)*	<0.001*
Pemphigoid	7	1.91 (0.77-3.94)	0.14
Pemphigus	1	1.75 (0.04-9.77)	0.92
*Pernicious anaemia*	*30*	*1.5 (1.01-2.15)*	*0.04*
Polyarteritis nodosa	2	4.11 (0.5-14.88)	0.15
Polymyositis	2	2.12 (0.26-7.67)	0.57
*Primary biliary cirrhosis*	*11*	*3.32 (1.65-5.96)*	<0.001*
Psoriasis	34	1.31 (0.9-1.84)	0.15
*Rheumatoid arthritis*	*157*	*1.63 (1.37-1.93)*	<0.001*
Scleroderma	6	1.9 (0.69-4.14)	0.19
Sjogren’s syndrome	5	0.89 (0.29-2.08)	0.96
*Systematic lupus erythematosus*	*28*	*4.78 (3.16-6.95)*	<0.001*
*Thyrotoxicosis*	*49*	*1.38 (1.02-1.84)*	*0.03*
Ulcerative colitis	31	0.87 (0.59-1.24)	0.49

*Significant P value after full Bonferroni correction = <0.00185.

Considering SAH that occurred at least a year after admission for immune-mediated disease, associations were similar as to those observed for the overall analysis (Table 2), except that autoimmune haemolytic anaemia, diabetes mellitus, myxoedema and pernicious anaemia lost significance (Table 4).

**Table 4:** **Rate ratios and 95**% **confidence intervals** (**CIs**) **for subarachnoid haemorrhage occurring at least a year after admission for immune**-**mediated disease**

**Immune mediated disease**	**Observed**	**Rate ratio (95% CI)**	**P value**
*Addison’s disease*	*18*	*1.82 (1.08-2.88)*	*0.02*
*Ankylosing spondylitis*	*34*	*1.55 (1.07-2.16)*	*0.01*
Autoimmune haemolytic anaemia	11	1.87 (0.93-3.34)	0.06
Chronic active hepatitis	1	0.21 (0.01-1.2)	0.14
Coeliac disease	49	0.89 (0.66-1.18)	0.48
*Crohn’s disease*	*133*	*1.32 (1.11-1.57)*	*0.002*
Dermatomyositis	2	1.11 (0.13-4.02)	0.83
Diabetes mellitus	390	1.10 (0.99-1.22)	0.067
Goodpasture’s syndrome	2	3.12 (0.38-11.27)	0.28
Hashimoto’s thyroiditis	8	0.97 (0.42-1.91)	0.93
*Idiopathic thrombocytopenia purpura*	*40*	*2.04 (1.45-2.78)*	<0.001*
Multiple sclerosis	87	1 (0.8-1.24)	0.97
Myasthenia gravis	8	0.79 (0.34-1.55)	0.61
Myxoedema	823	1.02 (0.95-1.1)	0.53
Pemphigoid	9	1.03 (0.47-1.96)	0.94
Pemphigus	2	1.21 (0.15-4.37)	0.91
Pernicious anaemia	66	1.22 (0.95-1.56)	0.12
Polyarteritis nodosa	2	1.15 (0.14-4.15)	0.85
Polymyositis	6	1.72 (0.63-3.75)	0.28
*Primary biliary cirrhosis*	*19*	*1.86 (1.12-2.9)*	*0.01*
*Psoriasis*	*122*	*1.35 (1.12-1.62)*	0.001*
*Rheumatoid arthritis*	*483*	*1.44 (1.31-1.58)*	<0.001*
*Scleroderma*	*19*	*1.83 (1.1-2.85)*	*0.012*
*Sjogren’s syndrome*	*34*	*1.94 (1.35-2.72)*	<0.001*
*Systematic lupus erythematosus*	*80*	*3.5 (2.77-4.36)*	<0.001*
*Thyrotoxicosis*	*153*	*1.38 (1.16-1.62)*	<0.001*
Ulcerative colitis	167	1.03 (0.88-1.2)	0.71

*Significant P value after full Bonferroni correction = <0.00185.

## Discussion and conclusion

Previous reports, combined with the results we present here, suggest that there may be an association between some immune-mediated diseases and the risk of subsequent SAH. Of note, both our study and the Swedish study of haemorrhagic stroke [5] found increased risks for ankylosing spondylitis, autoimmune haemolytic anaemia, Crohn’s disease, hyperthyroid conditions, idiopathic thrombocytopenia purpura, pernicious anaemia, psoriasis, rheumatoid arthritis and SLE. However, the Swedish study did not investigate the SAH subtype of haemorrhage, and indeed the mechanisms linking immune-mediated disease to SAH and other haemorrhage types may differ. Even if the fairly low levels of significant elevation of risk found in our study, such as those associated with myxoedema (at 1.15), are considered unimportant clinically, the high levels of risks associated with some diseases, for example SLE (at 3.76), are striking. This risk does not appear to be confined to recent hospital admission as the elevated risk for SAH remained for more than a year after being admitted for SLE.

Little is known about the cause of intracranial aneurysms or the process by which they form, grow, and rupture. The most common histological finding following an aneurysm is a decrease in the tunica media of the involved vessel causing defects of the normal arterial structure. These defects, combined with haemodynamic factors, lead to aneurysmal outpouchings at arterial branch points in the subarachnoid space at the base of the brain [10]. Spontaneous SAH is typically due to arterial bleeding and has been linked to hypertension, smoking and alcohol [11]. More rarely SAH is associated with vasculitis. We tested a wide variety of immune-mediated diseases with differing aetiologies in this exploratory study. The elevated risks may be a reflection of vasculitis and/or immune or inflammatory influences upon circulation. Most of the systemic autoimmune disorders linked to SAH in this study have been associated with vasculitis. These data therefore raise the possibility that the SAH is sometimes a manifestation of an associated focal vasculitis of the intra-cranial arteries. Without confirmation from pathology, this remains a hypothesis. Another more intriguing hypothesis is that berry aneurysms, or a proportion of them, are autoimmune and due to an inflammatory disease of blood vessels. This is partially supported by finding inflammatory infiltrates in the walls of cerebral aneurysms [12]. Only a few studies have investigated the possibility of some immunosuppressive medications commonly used in the treatment of immune-mediated diseases potentially contributing to an increased SAH risk. The use of non-steroidal anti-inflammatory drugs (NSAIDs) does not appear to be associated with an increased SAH risk [13]. More recently in a Taiwanese study, the use of a relatively high mean daily dose of steroid has however been proposed as an independent risk factor of increased SAH risk in SLE patients, but this needs to be confirmed or refuted in further work and in other immune-mediated diseases [14]. Further, the increased risks of SAH may have different causes in each disease. For example, type 1 diabetes (T1D) has recently been associated with a significantly increased risk of specifically non-aneurysmal SAH [15]. This is thought to be due to microvascular rather than macrovascular damage, which is known to occur in T1D through non-immune mediated mechanisms. The mechanism behind the association between SAH and diabetes mellitus may therefore, at least in part, differ from that of another immune-mediated disease.

A key strength of the dataset is its size with large numbers of fairly uncommon diseases. The risk of SAH was studied within a single population, using the same methodology, which means that direct comparisons of risk between different immune-mediated diseases can be made. The dataset has limitations. The data is based on prevalent cases of immune disease – the first recorded hospital admission or episode of day case care for each person with each condition – rather than being a cohort with follow-up from the date of first diagnosis. The dataset is limited to people who were admitted to hospital, or who received day case specialist care. This would not capture all people with each immune-mediated disease, although it should identify the great majority with subsequent diagnosed SAH. We lack treatment and disability data for the immune-mediated diseases; and we lack data on potential confounding factors such as detailed socioeconomic characteristics, ethnicity, smoking, alcohol intake and blood pressure. The effect of making multiple comparisons needs to be considered. It is possible that some of the associations that are significant at levels close to p < 0.05 may result from making multiple comparisons and the play of chance. This may particularly be so where there is no prior hypothesis to support the finding. On the other hand, even in a study with the number of comparisons that we have made, findings where the significance level is <0.001 or less, are unlikely to be attributable to chance alone. We studied 27 different immune-mediated diseases. At a significance level of p = 0.05, by chance alone one might expect one or two significant results. We have reported significant results for 15 of the 27: these associations with SAH are not the play of chance but a rather general characteristic of immune-mediated diseases.

Our findings and their interpretation should be regarded as speculative rather than definitive. The results represent what can be done using very large, linked, routinely collected administrative datasets; but such datasets lack detail. Alternative designs like epidemiological field studies - e.g. interviewing patients with SAH and controls about uncommon prior immune-mediated diseases, or following up cohorts of people with and without (say) SLE to identify later risk of SAH - would be substantial undertakings. Nonetheless, further work is needed, in different study designs, to confirm or refute the findings. Further studies should look at individual immune-mediated diseases in greater depth to aid understanding of mechanisms behind any association. This may be particularly warranted in people with diseases at relatively high risk of SAH, such as SLE.

## Competing interests

The authors declare no conflicts of interest.

## Authors’ contributions

MJG is the guarantor of and designed the study. OS and RG led the analysis. SVR and MJG contributed to the analysis and interpretation of the data. JP and LG added to interpretation of the results and to the manuscript. SVR wrote the first draft and all authors contributed to subsequent drafts and the final paper. All authors read and approved the final manuscript.

## Pre-publication history

The pre-publication history for this paper can be accessed here:

http://www.biomedcentral.com/1471-2377/13/176/prepub
